# A Study of Role Stressors and Job Satisfaction: The Case of MNCs in Collectivist Context

**DOI:** 10.3390/bs9050049

**Published:** 2019-05-07

**Authors:** Saif-ud-Din, Mohammad Ishfaq, Muhammad Imran Khan, Muhammad Asif Khan

**Affiliations:** 1 Department of HRM, College of Business, King Abdul Aziz University, Rabigh 21911, Saudi Arabia; 2 Department of Finance, College of Business, King Abdul Aziz University, Rabigh 21911, Saudi Arabia; mishfaq@kau.edu.sa; 3 Department of Management, Qurtuba University of Science and IT, Dera Ismail Khan 29050, Pakistan; rahi7228@yahoo.com; 4 Department of Marketing, College of Business, King Abdul Aziz University, Rabigh 21911, Saudi Arabia; mabkhan@kau.edu.sa

**Keywords:** role stressors, job stress, job satisfaction, mediation analysis, multinational corporation, collectivist context

## Abstract

Job stressors in organizational studies are commonly known as role stressors. These include role overload (RO), role conflict (RC), role ambiguity (RA) and job insecurity (JI). We explored the predicting role of these stressors on the overall level of job stress (JS) and job satisfaction (JSF). Moreover, we tested the role of JS as a mediator between the relationship of role stressors and JSF in a multinational corporation (MNC) in a non-western collectivist context (Pakistan). We obtained data through field surveys from 173 engineering employees from the electrical, mechanical, safety and chemical divisions. Role stressors significantly predicted overall level of JS and JSF. JS was also found to partially mediate the relationship between role stressors and JSF. The study findings suggest that foreign ownership needs to focus not only on the economic value, but also the organizational and job design to mitigate the detrimental role of selected stressors. The results of this study have important implications for MNCs in general, and in developing countries in particular. Theoretical and managerial implications are discussed with recommendations.

## 1. Background

Human resource management, planning, development and retention of employees are the core issues of an organizational success. In pursuit of organizational success, a stress free working environment is essential for the smooth running of the business. Stress is the pressure, force or tension that an incumbent experiences in the workplace and tries to resist in order to keep the working environment normal [1]. Studies have shown that JS is an international issue and the annual cost to organization and societies worldwide is estimated to be in the hundreds of billions of dollars due to stress consequences [2,3]. Stressors are multi-dimensional and the accumulation of stressors can be a foremost cause of JS resulting in poor JSF [4,5]. Attainment of a stress free workplace is an art of coping with stress. In developing nations like Pakistan, JS negatively predicts JSF, like in developed nations [6]. Organizations are a constellation of roles comprising a number of tasks assigned to individuals for achieving organizational expected targets [7,8]. However, roles predominantly reflect individual’s behavior, which is a result of the interaction between role sender and role incumbent [9]. 

The role incumbent in an organization meets the expectations of the role sender by investing his/her physical/mental resources and starts to perform his/her duty to become a part of the system through this role. People gain stress when their expectation is not met by the organizational resources due to excessive work compared to their physical/mental resources to tackle that assigned work [10]. Research revealed that improper role stressors management trigger individual stress symptoms at the workplace, which in turn, threaten individual behavior and JSF [11]. Recently researchers in cross cultural MNCs have focused on multiple factors that influence employees’ behaviors and attitudes due to their distinctive characteristics, such as global dispersion, and inherent cultural diversity and its dynamic structure [12].

Like other developing nations, the economy of Pakistan is presently in a transition process affected by multiple factors (for example, continuing high rate of inflation, political disputes (both internal and external), growing population and unemployment rates), where traditional employment patterns (long term) and job security are changed by more piece rate and temporary contracts [13]. We chose to study MNCs because they have several unique characteristics, such as organizational complexity, and use a distinctive set of organizational structures to pool together shared resources to transfer multifaceted and advanced knowledge and technology over a distance [14]. This distinctive structure is further employed to manage these demands, such as roles for foreign workers, and the distinctive group of managers and coordinating mechanisms among the international virtual teams. MNCs are well equipped with advance technology, complex organizational structure, and innovative products. Moreover, the product or services produced by MNCs are not merely required to satisfy local customers, but also the international markets, to meet the growing customer demands through different channels of investment, such as foreign direct investment (FDI). Given that, MNCs (owned by developed countries) have been springing up in developing countries in search for a new market for their products and services due to their abundance in natural resources and cheap labor.

Past research has explored that labor markets and work organizations are affected by globalization and exacerbates job characteristics such as job demands, JI, low job control, long work hours, psychosocial stressors [15]. These factors indeed make a workplace more challenging and stressful for both local and foreign workers which in turn, largely contribute to role stressors specifically the RO [3,16]. Hence, in MNCs context we need further understanding to identify the causes that trigger JS and reduce employee’s satisfaction. The collectivist philosophy is used to mediate this relationship. The most frequently used role stressors in the work place are RA, RC, and RO, which results in JS and job dissatisfaction [17]. Both RC and RA, alongside stress and RO, are clearly associated with low JSF [9]. The literature on this connection varies in different organizational contexts, for instance, RA was found to be more negatively related to JSF than RC, and the same with other role stressors [18].

In view of these challenging patterns of MNCs, the purpose of this study is to empirically examine the relationship between role stressors, JS and JSF in a MNC operating in Pakistan. This study is further motivated by: (a) the need to broaden the context of stress theory in non-western MNCs. (b) the need to examine the underlying process through which role stressors influence JSF by focusing on JS. (c) The desire for a more comprehensive understanding of JS as a mediator in relation to role stressors and JSF. (d) The opportunity to explore JI in non-western settings with an increased portfolio of role stressors in a desired MNC.

## 2. Theoretical Framework and Hypothesis

### 2.1. JS in Collectivist and Non-collectivist Societies

It is a known reality that much of the employees’ potential is being wasted due to JS in almost every organization. Stress is not confined in any geographic location, industry or profession, therefore the need is to assess its impact on different job related scenarios. Extensive work has been done to explore the relationship between role stressors, JS and its influence on employees attitude in the western individualist cultural context, whereas a little attention has been given to Asian collectivist countries [19,20]. Like in developed countries, stress in developing nations is also considered as a hidden threat at all level of employees in the organization [21]. On the other hand, it is important to focus collectivist cultures because MNCs increasingly relocating their operations in the developing countries [22,23]. 

Collectivist attitude among the organizational settings in developing countries suggest more complex dynamics mediated by one emotion towards the collectivity [20]. Spector et al., [24] study middle level managers in twenty-four nations from different organizations about RC, RA, and RO. They found a close cultural relevance of these role stressors. Hence, Individual experience stress not only in contemporary complex and globalized societies, but in developing societies as well [3,20]. Moreover, cultural patterns and several relations evolve in different ways in order to deal with different kinds of contextual and ecological stressors around the world [25].

#### 2.1.1. Relationship of Role stressors, JS and JSF

First hypothesis, measure role stressors, JS and JSF relationship in MNCs. Role stressors (a combination of four factors, i.e., RO, RC, RA and JI) have been a major concern at the workplace and considered a main source for job dissatisfaction [26]. Past studies found mix (positive and negative) relationships between role stressors, JS and JSF [17,27]. Conservation of Resource (COR) theory [10] further explain that stress leading causes results resource losses for example, workplace conflicts may drains individual’s resources, waste time, and distract them from their basic roles. This notion further asserts that stress arises from three conditions: (1) when individual’s key resources are threatened (2) when resources are lost, or (3) when a person fail to generate resources despite significant investments are made. 

Based on arguments above, stress cause losses that occurs due to stressors creating circumstances when individuals come under exceeding demand resulting psychological discomfort [28]. Moreover, Karasek, [29] job strain model have documented that the joint effects of job demand context results psychological strain specifically when the individual resource does not meet those demands. This model further predicts “job characteristics as the significant determinants of psychological strain”. The dimensions of role stressors are interconnected by three distinct constructs such as RO, RA, and RC [26]. We have included multiple measures in a model because it clarifies the variance nature of the predictors and criterion variables [30]. In our understanding all the behavioral outcomes of the role stressors are mediated by one’s overall level of JS caused by role stressors.

The first factor in the model *RO* has significant positive influence on JS [31] resulting poor JSF [17]. Empirical evidence have revealed that RO is associated to a variety of physiological and behavioral attitudes appearing in the form of JS, burnout, and job dissatisfaction [32]. A meta-analysis showed that, role stressors has direct negative link with JSF [33]. The second factor RC on the other hand is a result of difference of opinion between two or more employees of the organization causing by incompatible decisions [34]. In a RC situation individual may be caught between the crossfire of two managers and the desires of two operational groups in the organization [35]. Kahn et al. [9] observed that RC creates multiple adverse situations at the workplace producing high levels of conflicting situation and ambiguity which lead to job dissatisfaction. This notion was supported by other researchers and found that RC and RA are clearly associated with job dissatisfaction and dysfunctional behavior as a consequence of stress and anxiety [18]. In Asian context, RC positively and significantly related to job anxiety and JS among middle-level cadre of employees [5,27,36]. Thus, stressful environment exists where employees simultaneously perform multiple roles. 

The third factor RA situation is created at the work place ‘when individual has less information necessary to carry out his job properly’ [37]. Moreover, RA situation arises in organization due to lack of role clarity [9,18]. In a western context, RA is more stronger predictor of JSF than RC [38]. It is well documented in the literature that RA is a positive predictor of JS and job dissatisfaction [19,39]. The fourth factor JI added in the model is generally considered to be stressful and adversely influence individual’s performance [36], as well employees work health and wellbeing [40]. Probst and Lawler [41] report that compare to their western counterparts, employees with collectivist cultural values react more inversely to the threat of JI. JI is a potential threat to the continuity of one’s current job and become stressor for different work scenario [42]. JI is considered as uncertain because it signifies unpredictability and uncontrollability at workplace [43]... These arguments give us more confidence to test JI as a role stressor and a potential predictor of JS and JSF in this foreign ownership firm.

The second main hypothesis (H2) examines role stressors and JSF relationship. There is an ample evidence in the literature that JSF results from employees’ positive reaction about their specific role which occurs by comparing the actual results with the expected output, such as desired, wanted, needed, or perceived to be just and rational [17]. Increasing work burden and professional uncertainty inversely influence employees’ JSF [8]. Among the physical trainers in South Eastern European RC and RA are found significant predictors of JSF [44]. Role stressors has direct negative relation with JSF and vastly inevitable in nature because of their positive and negative relations with JS and JSF respectively [27,45]. Hence, we propose hypothesis H1 and H2 followed by the sub-hypothesis.

**H1:**  *Role stressors will be positively related to JS such that:*

*(H1a), RO will be positively related with JS, (H1b), RC will be positively related with JS (H1c), RA will be positively related with JS (H1d), JI will be positively related with JS.*

**H2:**  *Role stressors will be negatively related with JSF such that:*

*(H2a), RO will be negatively related with JSF (H2b), RC will be negatively related with JSF (H2c), RA will be negatively related with JSF (H2d), JI will be negatively related with JSF.*

#### 2.1.2. Mediating Role of JS

Job strain model [29] guides us that job characteristics are the major source of psychological strain, created by high job demands and low control over the resources. Individuals with inequality in job demands and resources given are less likely to respond effectively. These situations indeed threaten psychological well-being of workers and exacerbate individual stress level. This argument further predicts that work overload and time pressure can increase job strain. However, the extent to which RO, RC and RA contribute to stress under different working context has not yet been studied [3]. It has been reported that MNCs’ employees have high negative stress that lead to individual physical and psychological illness which in turn results low JSF [46]. Theoretically, role stressors by its job-related antecedents lead to organizational inequity results psychological strain and diminution of employee’s performance and satisfaction. Karasek’s [29] job strain model and the Social exchange imbalances [47], would support such mediation for JS where psychological stress jointly effected by job demands and the individual resources to meet those demands [3]. 

We offer that stressors differentially affect the employees overall level of JS which further negatively results individual JSF. JS was also observed as a partial mediator between workplace discrimination and JSF [48]. Moreover, the extent to which RA, RC, RO and JI contribute to JS and JSF has not yet been studied specifically under the non-western MNCs context. We therefore intend to explore the predicting role of stressors on JS and JSF to investigate the indirect path of this relationship. Therefore, we posit H3 and H4.

**H3:**  *JS and JSF are negatively related.*

**H4:**  *JS will mediate the relationship of:*

*(H4a), RO and JSF (H4b): RC and JSF (H4c), RA and JSF (H4d), JI and JSF*

**Figure 1:**
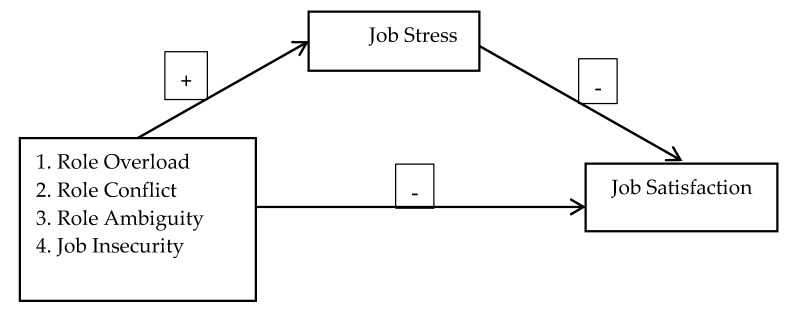
Empirical model of the study.

In the light of above discussion and hypotheses, we propose following model (Figure 1) to be tested in this study. 

## 3. Research Methodology

### 3.1. Sample and Procedures

Primary data were collected through a structured questionnaire from 173 employees of the organization with 97% response rate. The sample size 180 was determined based on statistical formula [49], from a known population of 314 employees. Convenience sampling technique has been used. 

The demographic information revealed that all the participants in the study consists of male because the engineering profession traditionally being a male dominant field specifically in Pakistan. The manpower included most of the engineers, sub engineers, and helpers. The average age of the respondents was 35 years. Maximum employees have less than five years of experience. Profession wise the sample consist of 32 engineers from Electrical department, 66 from Civil, 41 from Mechanical and rest of them from safety, chemical, and other Divisions.

### 3.2. Measures

The questionnaire survey was used as an instrument for data collection comprising two parts. Part A dealt with the demographics while Part B dealt with the research variables. We measured RO by using 11 item scale [50]. RC and RA were measured by using the scales developed by Rizzo et al., [18] consisting eight and six items respectively. Furthermore, 20 items of the JI were adapted from Ashford et al., [51]. JS was measured with 13 items from Parker, and DeCotiis [52]. Finally, JSF was measured with 14 items with 5-point likert scale [53]. All the items were measured on a 5 point likert scale. 

## 4. Results 

Correlations and descriptive statistics are provided in the following sections.

The mean, SD, reliability estimates and zero order correlations were used (Table 1). Results revealed that all the research variables are significantly correlated in expected direction. A Cronbach alpha value for each measure was above 0.70. Moreover, the correlation value of variables in the model ranged from a minimum correlation of −0.347 between RC and JSF to a maximum of 0.658 between RO and RC.

### 4.1. Tests of Hypotheses

#### 4.1.1. Role Stressors and JS (H1a–d)

The first step in a conceptual model, Figure 1, is to exhibit that role stressors are a positive predictor of JS. Hypothesis 1 is tested by the series of sub hypothesis (H1a-d) by entering role stressors one by one in the equation. As presented (Table 2, Step 1), role stressors significantly and positively predict JS. Therefore, results related to H1a conformed that RO was significant positive predictor of JS (β = 0.92, *p* <0.01, ΔR^2^ = 0.92, *p* < 0.01). Similarly, significant values were found for H1b, RC and job stress (β = 0.52, *p* < 0.05, ΔR^2^ = 0.31, *p* < 0.05), H1c, RA and job stress (β = 0.26, *p* < 0.01, ΔR^2^ = 0.23, *p* < 0.01), and H1d, job insecurity and job stress (β = 0.63, *p* < 0.01, ΔR^2^ = 0.35, *p* < 0.01). The obtained values for overall model were also significant. So, hypothesis 1, followed by sub hypothesis H1a to H1d was fully supported and fulfills the first criteria for mediation outlined by Baron and Kenny [54]. 

#### 4.1.2. Role Stressors and JSF (H2a–d)

The second step (Figure 1) exhibits that role stressors negatively predict JSF presented in Hypothesis 2 (H2a-d). By testing Hypothesis 2a, results in (Table 2, step 2) indicate that, RO negatively predicted JSF (β = −0.64, *p* < 0.01, ΔR^2^ = 0.26, *p* < 0.05). Similarly, *H2b*, RC and JSF (β = −0.25, *p* < 0.05, ΔR^2^ = 0.12, *p* < 0.05), *H2c,* RA and JSF (β = −0.20, *p* < 0.05, ΔR^2^ = 0.26, *p* < 0.05) and finally, H2d, JI also negatively predicted JSF (β = −0.37, *p* < 0.05, ΔR^2^ = 0.20, *p* < 0.05). The obtained values for overall model were also significant. So, hypothesis 2 followed by Sub-hypothesis H2a to H2d was fully supported and fulfills the second criteria for mediation outlined by Baron and Kenny [54]. 

#### 4.1.3. JS and JSF (H3)

The third step (Figure 1) exhibit that JS negatively predict JSF. Therefore, we test *Hypothesis 3* by entering JS in the equation. Results confirmed that JS negatively predicted JSF (β = −0.39, *p* < 0.01, ΔR^2^ = 0.26, *p* < 0.01). The overall model was also found significant F = 60.33, *p* ˂ 0.001). So, hypothesis 3 was also accepted.

The above main hypotheses followed by sub-sets proved that all the predictors i.e., role stressors are significantly related to JS (Mediator) and with JSF (criterion variable) as illustrated in Figure 1. Consequently, we achieved the first and second requirements for mediation analysis outlined by Baron and Kenny [54] mentioned in the following mediation analysis.

### 4.2. Mediation Test

Barron and Kenny [54] protocol has been used in this study for mediation analysis. Therefore, following conditions are necessary to be met to have mediation effect. First, Role stressors (the predictors) must be significantly related to JS (mediator). Second and third, role stressors and JS (mediator) must be significant predictors of the JSF respectively. Fourth, when both predictors and the mediator at the same time entered in a regression equation, the effect of each role stressors of the JSF must be insignificant. Furthermore, it is important to test the significance of the indirect path of each role stressors by means of JS. The indirect effect is denoted as the product of the X→M path (a) and M→X path b or ab. The standard error of the indirect effect (S_ab_) is given by Sobel, [55] as follows:

S_ab_ = √b^2^s^2^_a_ + a^2^s^2^_b_ + s^2^_a_s^2^_b_

where S_a_ and S_b_ are the standard errors of a and b path respectively.

The following (Table 3) presents the mediation analysis of *hypotheses 4* and its sub-set (H4a-d). 

#### 4.2.1. RO and JSF (H4a)

For testing of sub-hypothesis H4a, we expected that JS would mediate the relationship between RO and JSF. The RO was controlled in the first block of hierarchical regression in Table 3 and in the second block of the regression analysis, the mediator-JS was included into the model in step 3, it has significant effect on JSF β = −0.41, *p* < 0.01, ΔR^2^ = 0.33, *p* < 0.01). Although, the results remain significant but the effects of RO on JSF reduced from β = −0.64 to β = −0.41 as difference shown in Table 2 and Table 3. 

Moreover, Judd and Kenny [56] difference in coefficient approach were used to find the indirect effect. The value −0.23 of indirect effect was obtained by subtracting the un-standardized regression coefficient of predictors in step 3 (β = −0.41) from the un-standardized regression coefficient of dependent variables in step 2 (β = −0.64). The value of indirect effect (β = −0.23) fell under the bootstrap interval. For further confirmation of mediation of JSF, Sobel test was conducted by putting the values of un-standardized regression coefficients and standard errors of path “a” and path “b” of the model in the Sobel test (Auto calculator available online). The significant result *p* < 0.001 of the test was acquired. Therefore, partial mediation effect for RO and JSF was found. Sub-hypothesis H4a was therefore, partially supported.

#### 4.2.2. RC and JSF (H4b)

In sub-Hypothesis *H_4b_* we expected that JS mediates RC and JSF relationship. After controlling RC in the first block of hierarchical regression in Table 3, the mediator JS was entered into the equation model on the second block. The effect of RC changed from β= −0.25 to β= −0.06 with *p* < 0.01, depicted in Table 2 and Table 3. The inclusion of JS in block 2 in Table 3 reduced the effect of RC on JSF to the maximum level; however, the impact of JS remained significant thereby achieved partial mediation. The indirect effect of mediation was found as β= −0.19 which falls under the bootstrap interval and the result of the Sobel test was also having *p* < 0.01 as the same method mentioned in the result of sub-hypothesis H4a above. Therefore, sub-hypothesis H4b was partially supported.

#### 4.2.3. RA and JSF (H4c)

Sub-Hypothesis H4c posited that JS mediates RA and JSF relationship. The first and second condition of protocol was met in Table 2 as discussed above. In Table 3 (block 1) of the hierarchical regression process, the effect of RA reduced; though remain significant with addition of JS in the model in block 2. Hence, results proved partial mediation. The indirect effect was found β = −0.07 which falls under the bootstrap interval [56]. The result of the Sobel test was also found significant. So, sub-hypothesis H4c was also partially supported.

#### 4.2.4. JI and JSF (H4d)

Sub-Hypothesis H4d posited that JS mediates JI and JSF relationship. After controlling the effect of JI in the first block of hierarchical regression equation in Table 3, the mediator JS inserted into the model in block 2. The effect of JI reduced from β = −0.37 to β = −0.19 with *p* < 0.01 given in Table 2 and Table 3. The inclusion of JS in block 2 in Table 3 reduced the influence of JI on JSF, however, impact of JS remained significant which meets the conditions of partial mediation. The indirect effect of mediation was found as β = −0.18 through the same method as recommended by Judd and Kenny [56], which falls under the bootstrap interval, the result of the Sobel test was also remained significant. Therefore, sub-hypothesis H4d was partially supported.

## 5. Discussion

This study examines the relationship between role stressors, JS and JSF with the main objective of establishing whether JS can play the role as a mediator between the selected stressors and JSF. We explored the cross cultural applicability of stress models in non-western culture like Pakistan. This research further demonstrated the relationship of role stressors with JS and JSF with increased portfolio in role stressor model by adding JI in a framework. We argue that JI causes relatively stress among the developing countries’ employees alike in developed nations. Our findings confirmed that role stressors significantly influence JS and JSF both directly and indirectly. Secondly the relationship of each role stressor with JS is significantly positive. This is in the line with previous studies where two role stressors i.e., RA and RC were positively associated to work stress [17,57]. 

The mean value for JI in this research is slightly less than the neutral meaning that employee in this MNO feel less JI. These results are contrary to the western views, where results indicated that ‘collectivist values were more likely to be affected by JI than individualist counterparts’ [41]. Hence, individual differences with reference to collectivity are countable to explore JI in developing countries. On the other hand our results confirmed that, JI predicts JS and JSF in collectivist context in line with the past studies conducted in individualistic context [40,41].Furthermore, the mean score for JSF is also higher which might play the role to alleviate the feelings of JI. 

This study proved that role stressors have significant and direct negative relationship with JSF. This is in conformity with those of the previous studies and showed that employees while facing these stressors become dissatisfied from their jobs [44,58,59]. Moreover, MNCs in Pakistan running their businesses under immense pressure of competition with local and other MNCs, which have a negative influence on employee’s well-being and their JSF [40,60]. Results also confirmed that JS significantly and negatively related with JSF which is in the line with other studies where JS causes job dissatisfaction [9,61]. Our study in the same domain also established that JS mediates the relationship between role stressors and JSF however, only partial mediation was proved. Moreover, the mediating role of JS in relation between RC and JSF was proven stronger because the coefficient of RC sufficiently reduced along with significance value (Table 3, step 3). It demonstrates that JS is a strong mediator for RC and JSF in comparison with other role stressors. This is also in the COR views as well [62]. In addition, the magnitude of research variables in a framework is diverse which demonstrate that the perception about role stressors, JS and JSF is different due to individual differences among the employee. 

### 5.1. Limitations and Future Researches

This study has also some potential limitations. One of the potential weakness of this research is the use of a cross sectional design, so we were unable to determine causality of the dependent and independent variables. Therefore, we could not analyze whether role stressors cause a feelings of job dissatisfaction, nor could we test whether role stressors and JS negatively impact on JSF. Hence, a longitudinal research design could have been useful to determine causality. Another potential limitation needs proper attention from the researchers, for example, the relative closeness of predictors (role stressors) and the mediator (JS). This might be sometime creates the issue of multicollinearity because role stressors measures may include items that capture strain due to stressors impact on criterion variables. Hence, it is recommended to check whether some measures unfortunately capture stressors and strain at a time in order to avoid common method bias and ensure the scale reliability. So, researchers in the future should pay more attention to the validity issues specifically in stressors and JS relationship, however, literature shows that role stressors effect JS with variance values [3]. 

Moreover, the collected data were based on self-reported assessments and the observed relationships may be inflated due to common-method bias. Thus, future studies should examine whether the relationship remains constant by using longitudinal or experimental research design and could address the problem of common method bias. Another limitation is the characteristics of the sample. The study was conducted in a single MNC which restricts its generalizability. 

Previous literature affirmed the direct relationship of role stressors and JSF in a meta-analysis see for example [33]. So, more theoretical support is required to conceptualize JS as a mediator between role stressors and criterion variables like JSF, organizational citizenship behavior, organizational commitment, and Turnover intention in different occupation. 

Hence, it would be more interesting to design a comparative study presented the cross cultural perspective about the mediating role of JS. We have selected JI to enhance the portfolio of role stressors to improve the model generalizability. Therefore, researchers might take benefits in the same domain to compare the model between western (individualist) and non-western (collectivist) MNCs context. In recent past researchers and practitioners are getting immense interest in this connection to explore the antecedents of JI across the cultures [41,63].

### 5.2. Recommendations

Our results showed that JS partially mediated between all the predictive variables and outcome variable. The findings validate the viability of social exchange theory [47] and COR view [62], where social exchange and resource imbalances unfold individual resources. Our study supports differential role of job stressors in predicting overall JS and JSF. The findings have practical implications such as role stressors and JS should be given adequate importance in MNCs as it may harm and infuse unpredictable and uncontrollable behavior among employees. Organizations therefore, should reduce negative consequences of role stressors or at least mitigate by using fair and open communication towards organizational change and other concerned policies. 

RO is another complex form of RC [9], the relationship may not be linear rather non-linear such as its role may be constructive in the early stages and may affect negatively in later stages. Supportive strategies such as creation of supportive organizational environment, role clarity, supervisory support, focus on job enrichment and job design, and planning career paths may help in coping against RC and other stressors.

The partial mediating role of JS suggests that in addition to direct effects of role stressors, MNCs should focus on JS in reducing the overall effect of role stressors on JSF to ensure a stress free environment and better work life quality to enjoy organizational effectiveness. We propose particularly in collective contexts that MNCs should promote open and direct communication with employees through training sessions (e.g., employee assistance programs ‘EAP’) to understand their psychological and emotional problems. In addition, try to resolve the issues when they arise as it may cause mental health issues [64] if problem persists. 

In sum, this study provides better alternative measures of reducing role stressors to examine employees’ work related environment in improving JSF. Our study contributes in extending JS models in collectivist context, and further, explores directly and indirectly important work behavior (JSF) affected by role stressors. By exploring COR and social exchange theories in non-western collectivist context, this study highlights the importance of cultural differences in organizational behavior in general and MNCs in particular. 

## Figures and Tables

**Table 1:** The correlation, mean and standard deviation of the variables.

Variables	RO	RC	RA	JI	JS	JSF	Mean	SD	α
RO	1						3.84	0.288	0.71
RC	0.658 **	1					3.89	0.510	0.71
RA	0.582 **	0.447 **	1				4.67	0.872	0.79
JI	0.445 **	0.351 **	0.364 **	1			2.89	0.442	0.84
JS	0.560 **	0.563 **	0.479 **	0.596 **	1		3.34	0.474	0.81
JSF	−0.506 **	−0.347 **	−0.475 **	−0.451 **	−0.511 **	1	4.10	0.367	0.87

Notes: * *p* < 0.05, ** *p* < 0.01; SD = standard deviation; α = alpha.

**Table 2:** Simple regression coefficients for each individual predictor.

Independent Variables	Step 1			Step 2		
	JS			JSF		
	B	F	R^2^	B	F	R^2^
Role Overload	0.92 **	78.29 **	0.31	−0.64 **	59.98 **	0.26
Role Conflict	0.52 **	79.51 **	0.31	−0.25 **	23.46 **	0.12
Role Ambiguity	0.26 **	50.83 **	0.23	−0.20 **	49.91 **	0.26
Job Insecurity	0.63 **	94.17 **	0.35	−0.37 **	43.57 **	0.20
Job Stress				−0.39 **	60.33 **	0.26

Notes: * *p* < 0.05, ** *p* < 0.01.

**Table 3:** Hierarchical regression for each individual predictor.

	Predictors	Step 3						
		JSF						
		B	F	R^2^	R^2^ (from Step 2)	∆R^2^	Bootstrap Interval	Sobel Test *p*-Value
Block-1	RO	−0.41 **	42.15 **	0.33	0.26	0.07 **	−0.38 to −0.13	0.000
Block-2	JS	−0.25 **						
Block-1	RC	−0.06	30.80 **	0.26	0.12	0.14 **	−0.27 to −0.11	0.000
Block-2	JS	−0.36 **						
Block-1	RA	−0.13 **	41.85 **	0.33	0.23	0.10 **	−0.12 to −0.05	0.000
Block-2	JS	−0.28 **						
Block-1	JI	−0.19 **	35.38 **	0.29	0.20	0.09 **	−0.28 to −0.11	0.000
Block-2	JS	−0.29 **						

Notes: * *p* < 0.05, ** *p* < 0.01.
